# Vaccine Hesitancy: Lessons Learned and Perspectives for a Post-Pandemic Tomorrow

**DOI:** 10.3390/vaccines10040551

**Published:** 2022-04-01

**Authors:** Francesca Scognamiglio, Davide Gori, Marco Montalti

**Affiliations:** 1 School of Hygiene and Preventive Medicine, Department of Biomedical and Neuromotor Sciences, Public Health and Medical Statistics, University of Bologna, Via San Giacomo 12, 40126 Bologna, Italy; frances.scognamigli5@studio.unibo.it (F.S.); marco.montalti7@studio.unibo.it (M.M.); 2 Unit of Hygiene, Department of Biomedical and Neuromotor Sciences, Public Health and Medical Statistics, University of Bologna, Via San Giacomo 12, 40126 Bologna, Italy

Infectious diseases have always posed a significant threat to mankind, resulting in high mortality and morbidity throughout history [1]. Currently, the burden of communicable diseases still remains very high in all age groups worldwide [2]. Moreover, the current COVID-19 pandemic has once again underlined the tremendous impact of transmittable diseases in terms of public health [1].

From this point of view, vaccines have always represented a fundamental public health tool. Indeed, mass immunization campaigns save millions of lives each year [3]. Furthermore, COVID-19 vaccines are enabling us to overcome the ongoing pandemic. Therefore, it is of utmost importance to promote vaccine uptake and to identify and address barriers for achieving adequate vaccination coverage.

Among the obstacles to reaching adequate vaccination coverage, vaccine hesitancy (VH) is rightfully included.

VH is defined by SAGE working group as “the delay in acceptance or refusal of vaccination despite the availability of vaccination services” [4] and was included by the World Health Organization (WHO) amongst the major health concerns in 2019 [5]. Going into detail, VH appears to be complex and context specific, varying across time, place and types of vaccines [4]. All these factors have been studied with respect to VH in recent decades and have played a big role during the COVID-19 pandemic and will certainly continue to do so in the post-pandemic future.

In the last two years, increasing attention has been paid to the determinants of vaccine uptake and vaccine hesitancy with respect to COVID-19 vaccines. In a recent systematic review of the literature, the authors investigated vaccine acceptance rates and determinants of vaccine hesitancy towards the current COVID-19 vaccination campaign globally [6]. The results showed that VH is a context-specific phenomenon, which varies according to geographical, social and demographic contexts [6]. Different population subgroups may have a greater tendency toward vaccine hesitancy and need to be targeted with specific strategies to counteract the phenomenon [6]. Of great concern is vaccine hesitancy in particular groups, such as healthcare workers, both because of their proximity to the patient and their health literacy role [6].

Other subgroups of particular interest are people with specific conditions that place them at greater risks of serious disease outcomes (e.g., people with comorbidities, pregnant women [7]). While people with comorbidities generally have higher vaccine uptake [8], some subgroups seem to present pockets of resistance (black adults with HIV, adults undergoing chronic dialysis and people addicted to drugs) [6]. These data can only be explained in the light of a complex analysis that also includes socio-economic and cultural factors.

In another study, an interesting insight into vaccine hesitancy in the population of the Veneto region in Northern Italy is provided [9]. In line with recent evidence [8,10], data collected show a difference by gender (women are more hesitant than men) and by age group (people aged between 30 and 70 years are more hesitant than people of other age groups) [9]. The difference in the distribution of vaccine hesitancy by age could be related to different exposure to social media [11], which has been identified as an important factor determining skepticism against COVID-19 vaccination. Some subgroups such as supermarket and shop workers were identified as more hesitant [9]. In addition, a positive association was found between level of education and vaccine acceptance [9]. People who had been vaccinated for flu had higher vaccination acceptance rates also for COVID-19 vaccines [9].

A study conducted in the cities of Palermo and Bologna investigating attitudes toward flu vaccination and COVID-19 vaccination reported results in line with previously presented studies [12]. In fact, in this study, vaccine hesitancy shows a different distribution according to gender and age (women are more hesitant than men, and people under 30 are less hesitant) [12]. Moreover, a lower educational level correlates positively with VH [12]. Another finding is that the propensity to become vaccinated against flu again correlates positively with the willingness to receive COVID-19 vaccinations [12].

Following these considerations, how the recent pandemic has affected the vaccine uptake of other types of vaccines should be considered. In a study conducted in China, the uptake of flu and pneumococcus vaccines was compared before and after the pandemic outbreak [13]. The results of this study show that the uptake of these vaccines increased during the pandemic, possibly due to an increased perception of susceptibility to infections among the population [13]. However, coverage was still suboptimal and specific strategies need to be developed to achieve adequate levels of vaccine coverage [13].

In the post-pandemic future, achieving and maintaining optimal influenza vaccine coverage will be of utmost importance. To achieve this goal, children are increasingly being vaccinated, including the use of innovative nasal vaccines [14]. With regard to childhood vaccinations, a factor influencing vaccine uptake should, therefore, be the VH of parents. In a recent study conducted in Khartoum state, Sudan, the authors showed that parental vaccine hesitancy directly influences measles vaccine uptake [15]. In another study conducted in the same state, the determinants of parental VH were investigated [15]. In addition to an exposure to misinformation and parental perceptions of vaccine effectiveness, the number of children, the age of the mother and barriers to access were identified as important determinants of parental VH [16]. The pandemic crisis has not only highlighted the need to address VH once again among parents/guardians but has also caused some delays in the schedules of children and adolescents for other types of vaccine.

For example, one vaccination that suffered a dramatic reduction in uptake in many countries during the pandemic is HPV vaccination. In one study, the role of health literacy in improving vaccine uptake was analyzed [17]. The results show that adequate communication and technical information aimed at debunking false believes (such as the fear that HPV agent may mutate back to a virulent HPV) can improve vaccine uptake [17].

Among the strategies aimed at improving vaccine uptake, the introduction of mandatory childhood vaccinations might be included. In a recent systematic review, the authors analyzed the impact of the pandemic on vaccination coverage and the incidence of vaccine preventable diseases, together with the implementation of mandatory vaccination in some countries of the WHO European Region [18]. Data collected show how the introduction of mandatory childhood vaccinations reduces the incidence of vaccine-preventable diseases and increases vaccine uptake [18].

Based on all the above considerations, the post-pandemic future looks like a challenging time that will require an additional public health effort to bring it back to its former level and to be prepared for any challenges that will emerge. Knowledge and the ongoing study of vaccine hesitancy remain the tools on which to base strategies to appropriately counter this phenomenon.
